# *BRAFV600E*-dependent Mcl-1 stabilization leads to everolimus resistance in colon cancer cells

**DOI:** 10.18632/oncotarget.10277

**Published:** 2016-06-24

**Authors:** Kan He, Dongshi Chen, Hang Ruan, Xiangyun Li, Jingshan Tong, Xiang Xu, Lin Zhang, Jian Yu

**Affiliations:** ^1^ Department of Pathology, University of Pittsburgh School of Medicine, Pittsburgh, PA 15213, USA; ^2^ University of Pittsburgh Cancer Institute, University of Pittsburgh School of Medicine, Pittsburgh, PA 15213, USA; ^3^ Department of Pharmacology and Chemical Biology, University of Pittsburgh School of Medicine, Pittsburgh, PA 15213, USA; ^4^ The Third Military Medical University Daping Hospital, Daping, Yu Zhong District, Chongqing 400042, P.R. China

**Keywords:** mTOR, everolimus, Mcl-1, BRAF V600E, ERK

## Abstract

mTOR activation is commonly caused by oncogenic mutations in RAS/RAF/MAPK and PI3K/AKT pathways, and promotes cancer progression and therapeutic resistance. However, mTOR inhibitors show limited single agent efficacy in patients. mTOR inhibitors suppress tumor cell growth and angiogenesis, and have recently been shown to induce death receptor/FADD-dependent apoptosis in colon cancers. Using a panel of *BRAF V600E* and WT colorectal cancer cell lines and *in vitro* selected resistant culture, and xenograft models, we demonstrate here that *BRAFV600E* confers resistance to mTOR inhibitors. Everolimus treatment disrupts the S6K1-IRS-2/PI3K negative feedback loop, leading to *BRAF* V600E-dependent activation of ERK and Mcl-1 stabilization in colon cancer cells, which in turn blocks the crosstalk from the death receptor to mitochondria. Co-treatment with inhibitors to Mcl-1, PI3K, RAF or MEK restores mTOR inhibitor-induced apoptosis by antagonizing Mcl-1 or abrogating ERK activation in *BRAFV600E* cells. Our findings provide a rationale for genotype-guided patient stratification and potential drug combinations to prevent or mitigate undesired activation of survival pathways induced by mTOR inhibitors.

## INTRODUCTION

The PI3K/AKT/ mTOR pathway is one of the most deregulated pathways in human cancer. To date, only a few PI3K-targeted drugs have emerged from clinical trials. Allosteric mTOR inhibitors such as Rapamycin derivatives or rapalogs are approved for clinical use in limited cancer types, while a variety of less selective ATP-competitive mTOR, PI3K or AKT or duel mTOR/PI3K multikinase inhibitors are still in clinical development [1, 2]. Everolimus (RAD001) is an orally available derivative of Rapamycin that binds to the mTOR cofactor FKBP12, to inhibit mTORC1 complex as well as mTORC2 complex upon prolonged or high dose exposure [1]. Everolimus is FDA-approved for patients with advanced renal cell carcinoma and currently in numerous clinical trials for advanced solid tumors including colon cancer [3]. Exceptional responses to Everolimus were reported in a few patients whose cancers harbor rare activating mutations in mTOR or loss of function mutations in its negative regulators TSC1/2 [4, 5]. The response to mTOR inhibitor mono therapy is generally limited, and a number of resistance mechanisms have been proposed [2, 6]. The best known is the disruption of a negative feedback loop upon mTORC1 inhibition which leads to AKT activation through upregulation of receptor tyrosine kinases or substrates such as the IGF-1R/IRS-1 axis [7, 8]. However, underlying genetic causes of differential response to mTOR inhibitors remain largely undefined.

RAS/RAF/MEK/ERK is another frequently deregulated pathway in human cancer and coexists with mTOR activation. Nearly half of all colon cancers contain *KRAS/BRAF* mutations and the numbers are higher in bigger or more advanced tumors. *BRAFV600E* is by far the most common *BRAF* activating mutation in colorectal cancers [9], and associated with several distinct clinic-pathological parameters, such as proximal location, mucinous histology, microsatellite instability (MSI), female gender, higher age and grade, and poor prognosis after failure of standard chemotherapeutic regimens [10, 11]. *BRAFV600E* selective inhibitors such as Vemurafenib (PLX4032) and dabrafenib (GSK2118436) are FDA-approved for the treatment of unresectable or metastatic melanoma. However, the response rate in metastatic colorectal cancer harboring *BRAFV600E* mutation is rather disappointing while the underlying mechanisms are not well understood [11–13], and the unresponsiveness might be caused by feedback activation of EGFR signaling [14]. These findings demonstrate that the efficacy of pharmacological targeting of an oncogenic driver is strongly influenced by cancer- or cell type-specific signaling. The role of mutant *BRAF* in mTORi response has not been determined.

Apoptosis induction is an important mechanism of anticancer agents including targeted therapies [15, 16]. The intrinsic apoptotic pathway is triggered by DNA damage or growth factor deprivation and regulated by the Bcl-2 family of proteins and mitochondria [17]. The extrinsic pathway is activated upon clustering of death receptors such as DR5 and assembly of death-inducing signaling complex (DISC) and caspase-8 processing. In some cells, caspase-8-dependent cleavage of Bid is required to amplify apoptotic signaling through the mitochondria to induce apoptosis [18]. Anti-proliferation and anti-angiogenesis activities of Rapalogs have been well-established [1, 2], and our recent work demonstrated that activation of ER stress and the DR5/FADD-dependent apoptosis contributes significantly to their therapeutic response in colon cancer cells and xenografts [19]. In this study, we uncovered a *BRAF*V600E-dependent mechanism underlying intrinsic and acquired resistance to mTOR inhibitors. These findings provide potentially useful biomarkers to help better design clinical trials and rational drug combinations to circumvent resistance.

## RESULTS

### *BRAF* (V600E) colorectal cancer cells are resistant to mTOR inhibitors

**Figure 1:**
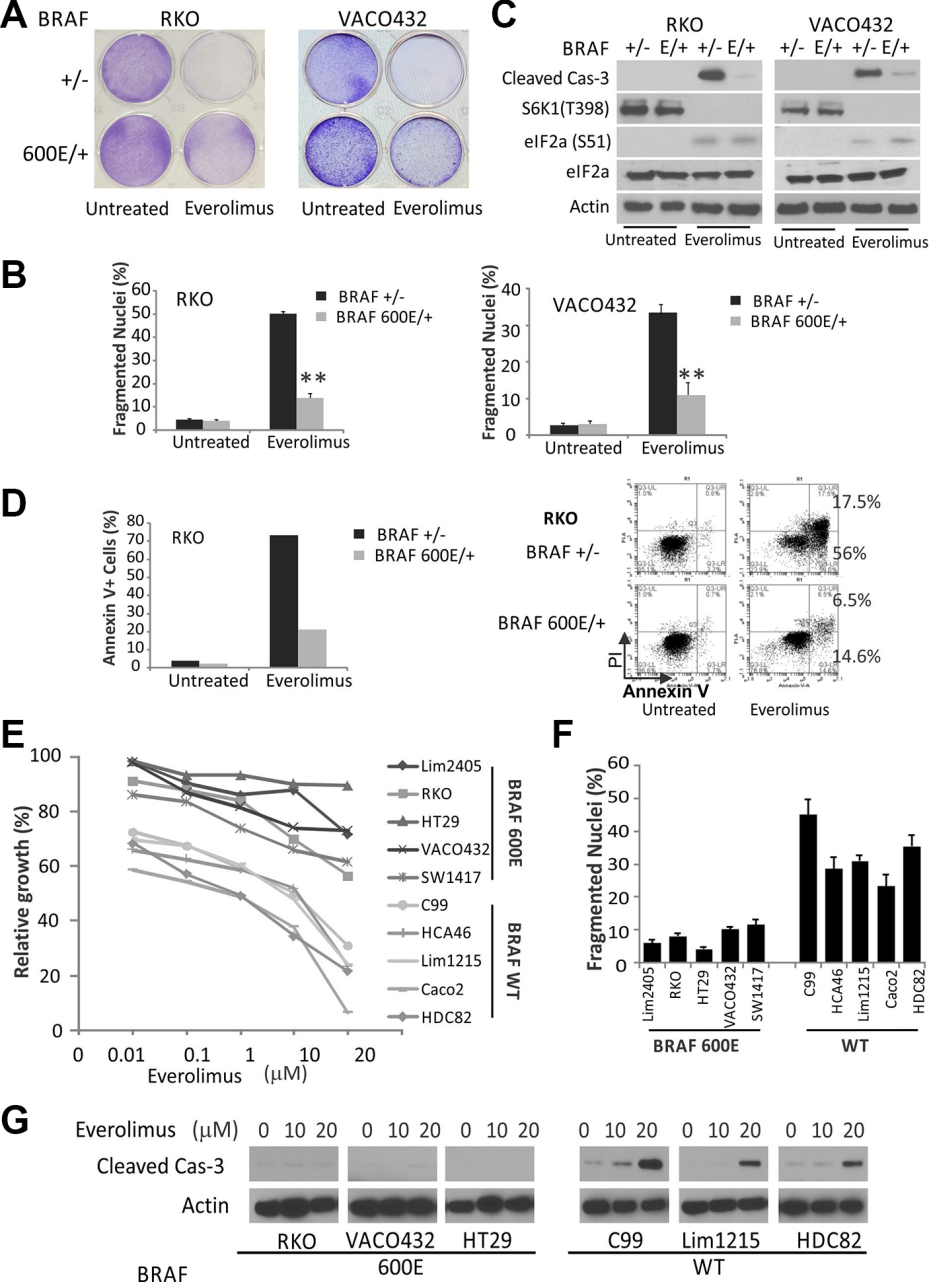
*BRAF V600E* colon cancer cells are resistant to Everolimus (**A**) isogenic pairs of BRAF WT and V600E (E) RKO and VACO432 cells were treated with 20 and 25 μM Everolimus, respectively. Attached cells after 48 h were stained by crystal violet. (**B**) cells treated as in A were analyzed for apoptosis by counting condensed and fragmented nuclei. **P < 0.01, 600E vs. WT. (**C**) cells treated as in A for 24 h were analyzed by western blotting. β-Actin was used as a loading control. (**D**) cells were treated as in A, stained with Annexin V/propidium iodide, and analyzed by flow cytometry (Right). Left, quantitation of Annexin V+ cells. (**E**) the growth of 10 colon cancer cell lines was determined by MTS assay following 72 h treatment with varying doses of Everolimus (10 nM to 20 μM). (**F**) apoptosis was analyzed after 48 h of 20 μM Everolimus. (**G**) cells treated as in F for 24 h were analyzed by western blotting.

Commonly used colon cancer cell lines frequently contain mutations in *KRAS/BRAF* [20]. To study a potential role of mutant KRAS/*BRAF* in Everolimus response, we took the advantage of isogenic colon cancer cell lines with targeted disruption of WT or mutant *BRAF* alleles, or mutant *KRAS* knockin or knockout cells. Using two pairs of isogenic colorectal cell lines RKO and VACO432 with either WT (+/−) or mutant (600E/+) *BRAF* [21], we found that WT cells (+/−) are more sensitive to Everolimus-induced growth suppression. (Figure 1A). Resistance of *BRAF* (600E/+) cells was associated with a strong reduction in apoptosis, as measured by nuclear fragmentation, flow cytometry and caspase-3 activation (Figure 1C–1D). The sensitivity and apoptosis in *BRAF* 600E/− cells were similar to parental cells (600E/+) (data not shown). We also examined apoptotic responses to Everolimus in isogenic CRC cell lines with WT or mutant *KRAS* (G13D or G12V) [22, 23], and mutant *KRAS* appears less well associated with apoptosis resistance (Figure S1A).

We decided to focus on *BRAF600E*, and selected a panel of ten colorectal cancer cell lines with either WT or *BRAF600E*. These ten cell lines well represent other driver mutations in colon cancer such as *APC*, *β-catenin*, *PIK3CA*, *p53*, or *SMAD4*, but all have WT *KRAS* (Table S1). Remarkably, all five 600E cell lines were found to be more resistant than any of the five *BRAF* WT cells across a range of Everoliumus concentrations in growth assays (Figure 1E). Everolimus (10–20 μM) treatment induced 20–45% apoptosis and activation of caspase-3 in *BRAF* WT cell lines within 48 hours, which was strongly suppressed in 600E cell lines (Figure 1F).

Treatment of rapalogs activates ER stress and the death receptor pathway in colon cancer cells *in vitro* and *in vivo* [19]. Unexpectedly, induction of ER stress assessed by p-eiF2a, or DR5, or inhibition of the prototypic mTOR target S6K1 was similar in RKO, VACO432 cells irrespective of *BRAF* status (Figures 1C, S1B). Consistent with our previous findings [19], Everolimus at lower doses (50 nM to 1 μM) induced a slight and reversible growth inhibition in either *BRAF* WT or 600E cells, but no apoptosis. These doses fully inhibited p-S6K1, but unable to reduce p-4E-BP1, cap-dependent translation, or induce ER stress and DR5 (Figures S1C and S1D). These results demonstrate that *BRAF*600E is associated with intrinsic resistance to Everolimus-induced apoptosis in colon cancer cells without affecting the induction of ER stress or DR5.

### Mcl-1 upregulation in *BRAF600E* colon cancer cells blocks apoptotic signaling from the death receptor to mitochondria

**Figure 2:**
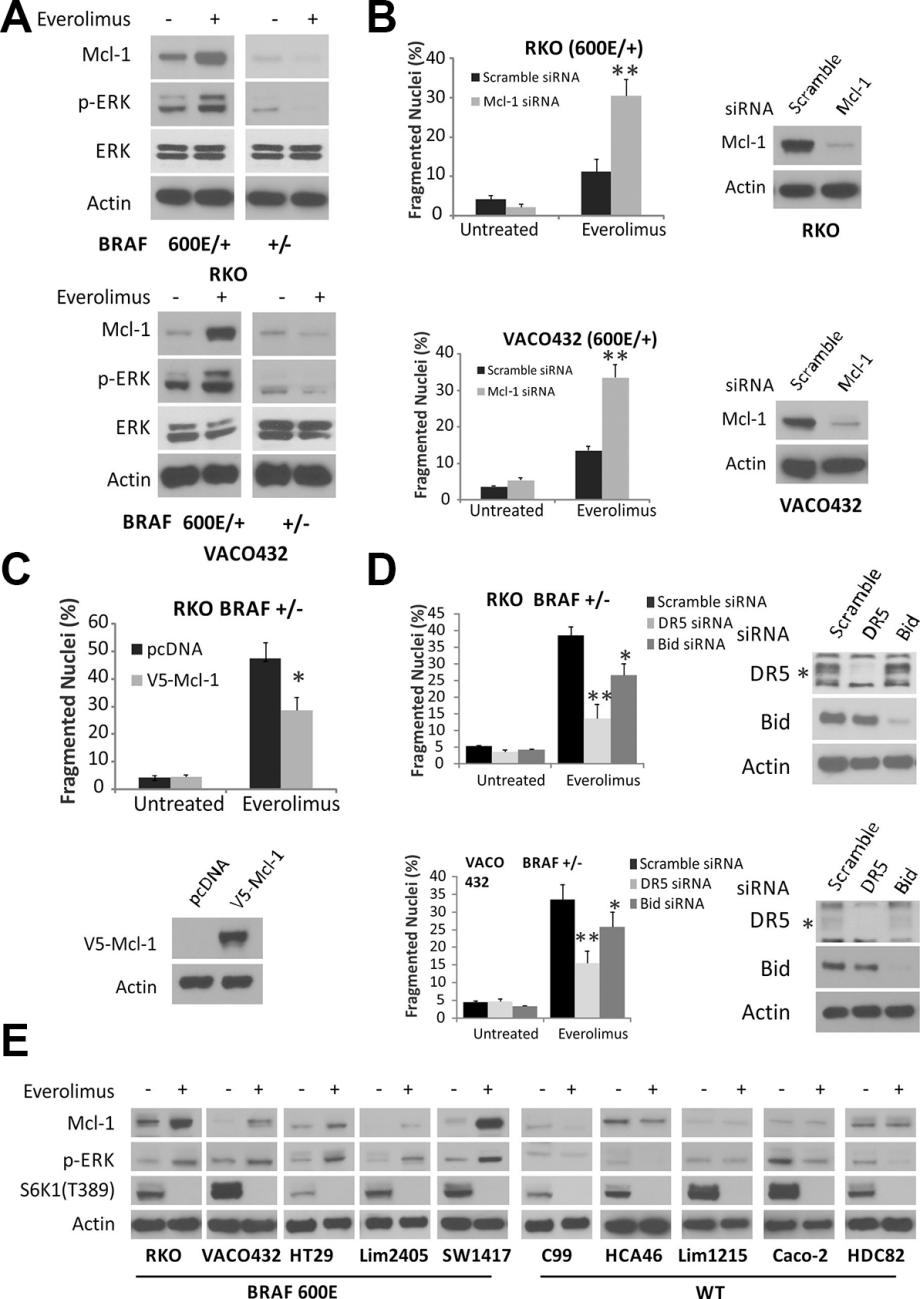
Elevated Mcl-1 in BRAF 600E cells inhibits Everolimus-induced apoptosis (**A**) isogenic RKO (top) and VOCO432 (bottom) cells were treated with 20 μM Everolimus for 24 h and analyzed by western blotting. (**B**) BRAF 600E RKO (Top) and VOCO432 (Bottom) cells were transfected with either scrambled or Mcl-1 siRNA for 24 h, then treated with Everolimus for 48 h, and analyzed for apoptosis. **P < 0.01, 600E vs. WT. (**C**) BRAF WT (+/−) RKO cells were transfected with either pcDNA or V5-Mcl-1 for 24h, then treated with Everolimus for 48 h and analyzed for apoptosis. (**D**) BRAF WT (+/−) RKO (top) or VACO431 cells (bottom) were transfected by either a scrambled, DR5, Bid siRNA for 24 h, treated with Everolimus for 48 h, and analyzed for apoptosis. **P < 0.01, *P < 0.05, V5-Mcl-1, DR5 and Bid siRNA vs. control. *DR5 specific band. (**E**) ten CRC cell lines with BRAF WT or 600E were treated with Everolimus for 24 h and analyzed by western blotting. B, C and D, apoptosis was analyzed by counting condensed and fragmented nuclei. Western blotting confirmed target depletion or overexpression. β-Actin was used as a loading control.

To investigate the mechanism of *BRAF600E*-mediated apoptotic resistance, we analyzed Bcl-2 family proteins including antiapoptotic members Bcl-2, Bcl-xL and Mcl-1, and proapoptotic members Bax, Bak, PUMA, NOXA, Bim and Bid. Everolimus treatment led to a strong induction of Mcl-1 in *BRAF600E* (*600E/+*) RKO and Vaco432 cells, associated with elevated p-ERK (Thr202/Tyr204). In contrast, Mcl-1 and p-ERK was reduced in *BRAF* WT cells (Figures 2A and S2A). Changes in other Bcl-2 proteins did not differ significantly between BRAF600E and WT RKO cells, while Bid cleavage was consistent with activation of the death receptor pathway (Figure S2A). Knockdown of *Mcl-1* restored Everolimus-induced apoptosis in 600E cells (Figure 2B). In *BRAF* WT (+/−) RKO cells, overexpression of Mcl-1 strongly inhibited Everolimus-induced apoptosis (Figure 2C), as well as knockdown of DR5 or Bid (Figure 2D). However, knockdown of other BH3-only proteins PUMA or Bim did not significantly affect Everolimus-induced apoptosis (Figure S2B and S2C).

We extended the analysis to ten CRC cell lines, and found Everolimus treatment-induced elevation in Mcl-1 and p-ERK in all five *BRAF*600E cell lines. In contrast, Mcl-1 and p-ERK were either reduced or unchanged in all *BRAF* WT cell lines. p-S6K1 was inhibited efficiently in all lines (Figure 2E). Compared to *BRAF* WT (+/−) RKO cells, *BRAF600E* cells (600E/+, 600E/−) cells were both resistant to apoptosis induced by the ATP-competitive mTOR inhibitor Torin-1, and exhibited elevated p-ERK and Mcl-1 (Figure S2D and S2E). These results suggest that Mcl-1 induction by mTOR inhibitors is associated with ERK activation in *BRAF600E* colon cancer cells, and blunts Bid-mediated crosstalk between the death receptor and mitochondria.

### *BRAF600E*-dependent activation of ERK leads to increased Mcl-1 stability upon the disruption of the S6K1-IRS-2/PI3K negative feedback by Everolimus

**Figure 3:**
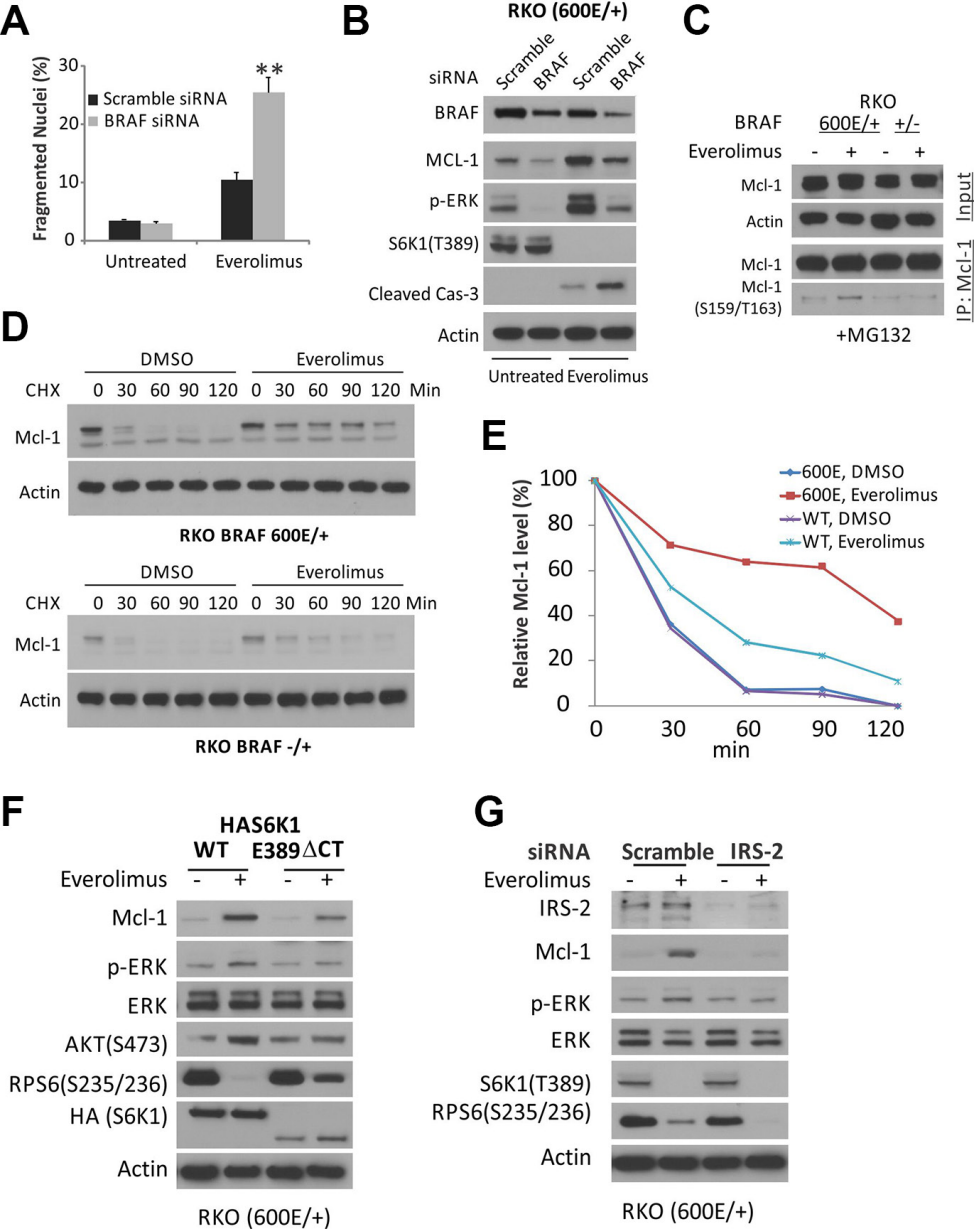
Everolimus treatment disrupts the S6K1-IRS-2/PI3K negative feedback and leads to MAPK activation and Mcl-1 stability in BRAF 600E cells (**A**) BRAF 600E RKO cells were transfected with either a scrambled, or BRAF siRNA for 24 h, treated with 20 μM Everolimus for 48h, and analyzed for apoptosis. **P < 0.01, BRAF vs. scramble siRNA. (**B**) cells treated as in A for 24 h and analyzed by western blotting. (**C**) isogenic RKO cells were treated with Everolimus for 6 h plus 5 μM proteasome inhibitor MG132 and 20 μg/ml Cycloheximide (CHX). Mcl-1 was immunoprecipitated (IP) and probed for p-Mcl-1. (**D**) isogenic RKO cells were treated with Everolimus and 20 μg/ml cycloheximide (CHX), and analyzed by western blotting. (**E**) relative Mcl-1 band intensity in D normalized to β-Actin compared to that of the t = 0 as 100%. (**F**) BRAF 600E RKO cells were transfected with either S6K1 (HAS6K1) or a constitutively active S6K1 (HAS6K1 E389 ΔCT) for 24 h, then treated with Everolimus for 24 h, and analyzed by western blotting. (**G**) RKO cells were transfected with either a scrambled, or IRS-2 siRNA for 24 h, then treated with Everolimus for 24 h, and analyzed by western blotting.

We then examined if *BRAF600E* is responsible for Everolimus resistance via Mcl-1 elevation. *BRAF* siRNA knockdown restored apoptosis and caspase-3 activation, and abrogated the elevation in p-ERK and Mcl-1 (Figures 3A, 3B, and S3A). Everolimus treatment did not increase, rather slightly decreased *Mcl-1* mRNA in *BRAF600E* cells, suggesting non-transcriptional mechanism for its elevation (Figure S3B). Mcl-1 is a fast turn-over protein, and contains PEST (proline, glutamate, serine, and threonine) motifs that are regulated by multiple kinases, including ERK, and targeted by ubiquitylation and proteasomal degradation [24–26]. The addition of proteasome inhibitor MG132 prevented Everolimus-induced decrease of Mcl-1 in *BRAF* WT cells, but strongly elevated p-Mcl-1 (Ser159/Thr163) in BRAF600E cells (Figure 3C), known ERK sites for inhibiting its degradation [24–26]. We then directly analyzed the half-life of Mcl-1. In the presence of translation inhibitor Cycloheximide (CHX), Mcl-1 levels sharply declined within 30 minutes in both WT and *600E* RKO cells (Figure 3D and 3E.). Mcl-1 decline was slowed down by Everolimus treatment substantially more in *600E* cells than WT cells (Figure 3D), with a half-life extended from 20 minutes to over 100 minutes (Figure 3E).

Everolimus (RAD001) treatment inhibited S6K-IRS-1 feedback, leading to PI3K-dependent ERK activation in some cancer cells [27]. To test if *BRAF600E* mediates ERK activation in colon cancer cells, we overexpressed the mTOR-insensitive and constitutively active form of S6K1 (HAS6K1 E389 delta CT) [28] and mTOR-sensitive WT S6K1 in 600E cells. The mTOR-insensitive S6K1 fully suppressed Everolimus-induced elevation in p-ERK and p-AKT, a PI3K target, and partially recovered p-S6 (Figure 3F). The levels of mRNA and protein of IRS-2, not IRS-1, increased significantly more in *BRAF600E* cells after Everolimus treatment (Figure S3C and S3D). *IRS-2* siRNA abrogated Everolimus-induced elevation in p-ERK or Mcl-1 (Figures 3G and S3A). Taken together, our results demonstrate that Everolimus treatment disrupts the S6K1-IRS-2/PI3K negative feedback in colon cancer cells and leads to *BRAF600E*-dependent activation of EKR and Mcl-1 stabilization.

### Overcome Everolimus resistance in *BRAF600E* cells

**Figure 4:**
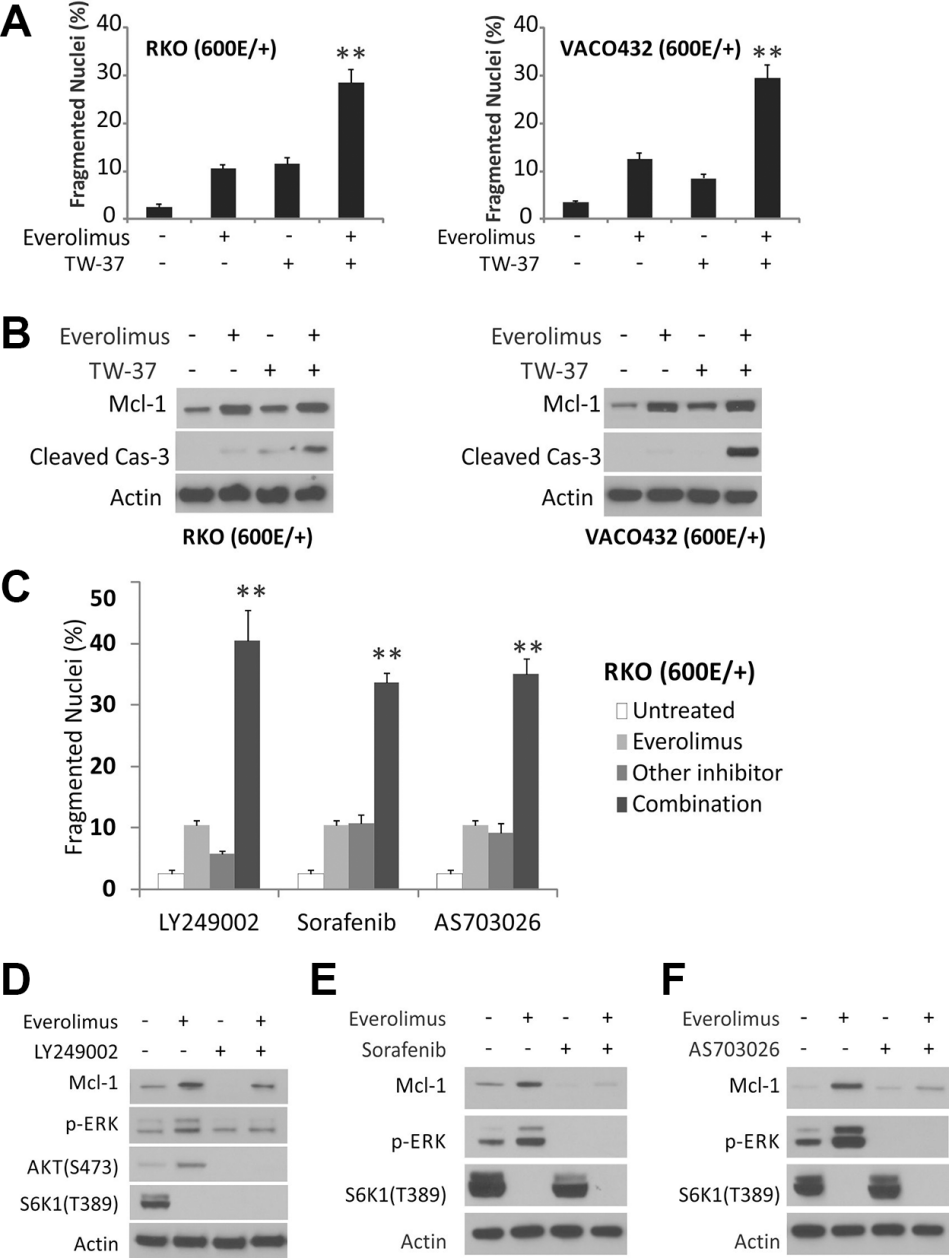
Pathway inhibitors restore apoptosis in *BRAF600E* cells (**A**) *BRAF 600E* RKO (Left) and VOCO432 (Right) cells were treated with 20 μM Everolimus, 5 μM TW-37, or their combination for 48 h, and analyzed for apoptosis. **P < 0.01, combination vs. single agent. (**B**) cells treated as in A for 24 h were analyzed by western blotting. (**C**) cells were treated with 20 μM Everolimus, 10 μM LY249002 (PI3Ki), 10 μM Sorafenib (RAFi) or 10 μM AS703026 (MEKi), or their combination for 48h, and analyzed for apoptosis. **P< 0.01, combination vs. single agent. The untreated and Everolimus bars are the same in each set. (**D**–**F**) cells were treated with indicated single agents or combinations as in C for 24 h and analyzed by western blotting.

The above findings demonstrate that *BRAF600E* mediates ERK activation and Mcl-1 stabilization downstream of PI3K, and predict that targeting any node in this pathway sensitizes colon cancer cells to Everolimus-induced apoptosis. The Mcl-1 inhibitor TW-37 [29] alone induced low levels of apoptosis in *BRAF600E* RKO and VACO432 cells, and was additive or synergic with Everolimus in apoptosis induction (Figure 4A and 4B). Inhibiting the upstream regulators using the PI3K inhibitor LY249002, RAF inhibitor sorafenib and MEK1/2 inhibitor AS703026, also restored Everolimus-induced apoptosis in BRAF600E RKO cells and prevented the induction of p-ERK and/or Mcl-1 (Figure 4C–4F). These data further confirm that PI3K/MEK/ERK-mediated Mcl-1 upregulation leads to Everolimus resistance in *BRAF600E* cells, which can be pharmacologically reverted.

### *BRAF600E* tumors are resistant to Everolimus and apoptosis *in vivo*

**Figure 5:**
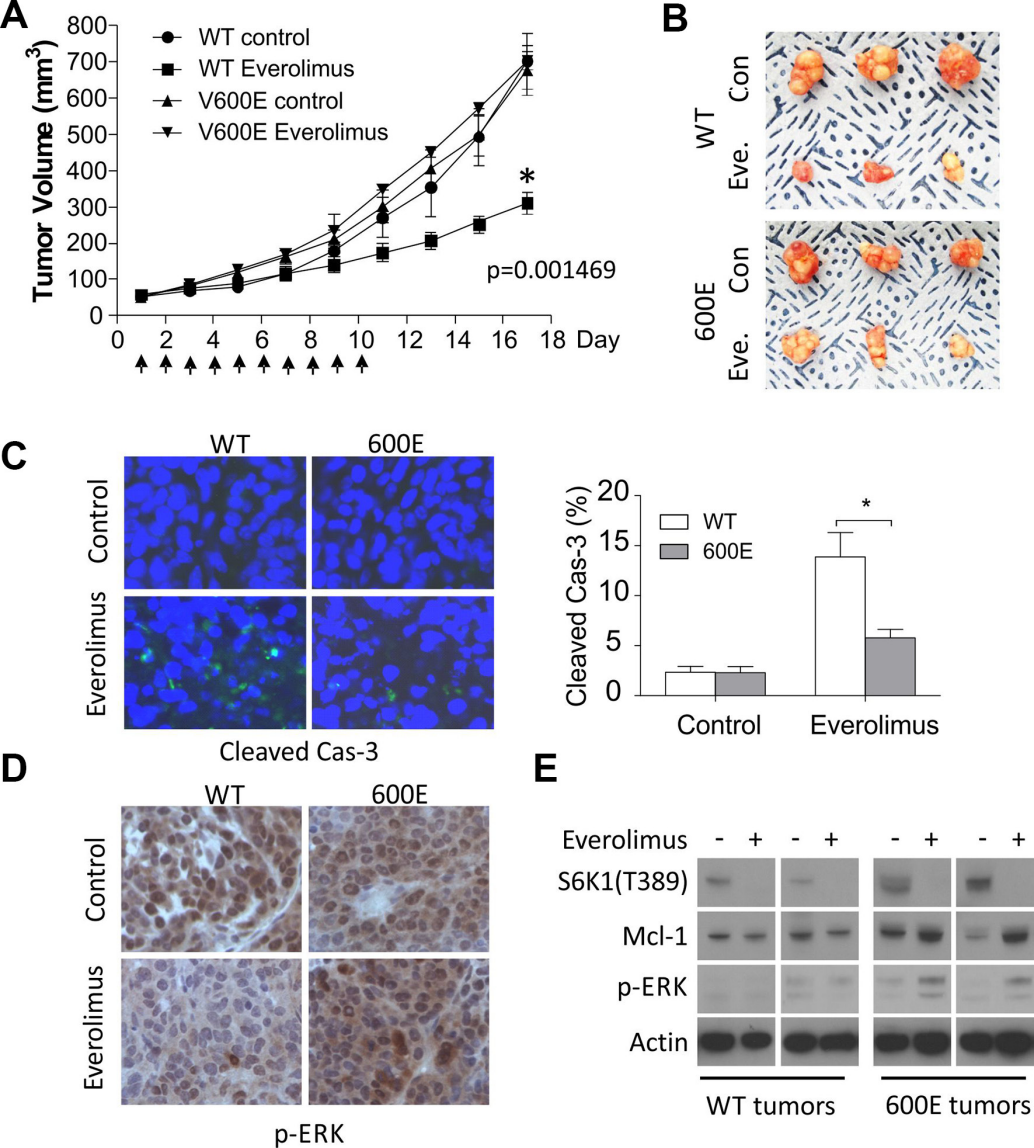
*BRAF 600E* tumors are resistant to Everolimus *in vivo* with reduced apoptosis and elevated p-ERK and Mcl-1 (**A**) *BRAF* WT or 600E RKO tumors were established in nude mice. Mice were randomized into two groups when tumors reach 50 mm^3^ to receive Everolimus (7.5 mg/kg/day) or vehicle for 10 consecutive days. Tumor volume was monitored 3 times per week and plotted. *N* = 8 mice/group. *P-value was calculated on the last measurements. (**B**) representative images of tumors at the end of experiments. (**C**) representative images of cleaved caspase-3 staining in tumors and quantitation (right). *P < 0.05, 600E vs. WT. (**D**) representative images of p-ERK staining in tumors. (**E**) two randomly chosen WT or 600E tumors from each group were harvested the day after the last treatment, and analyzed by western blotting.

To validate our findings *in vivo*, we established *BRAF* WT and 600E RKO xenografts in nude mice. Tumor bearing mice were randomized to receive either Everolimus or the vehicle for 10 days. Everolimus treatment inhibited the growth of WT tumors by 60%, but had little or no effect in 600E tumors (Figure 5A and 5B). Immunostaining indicated a significant reduction in activated caspase-3 and TUNEL staining in 600E tumors with elevated p-ERK (Figures 5C, 5D and S4A). Treatment-induced elevation in p-ERK and Mcl-1 was confirmed in tumor lysates by western blotting (Figure 5E). Consistent with cell culture data, inhibition of p-S6K1 and p-4E-BP1 and induction of ER stress were similar in WT and 600E tumors (Figures 5E, S4B and S4C). These data establish that BRAF600E impairs Everolimus-induced apoptosis *in vivo* via the p-ERK/ Mcl-1 axis.

### *BRAF600E* and Mcl-1 upregulation in Everolimus-resistant cultures selected *in vitro*

**Figure 6:**
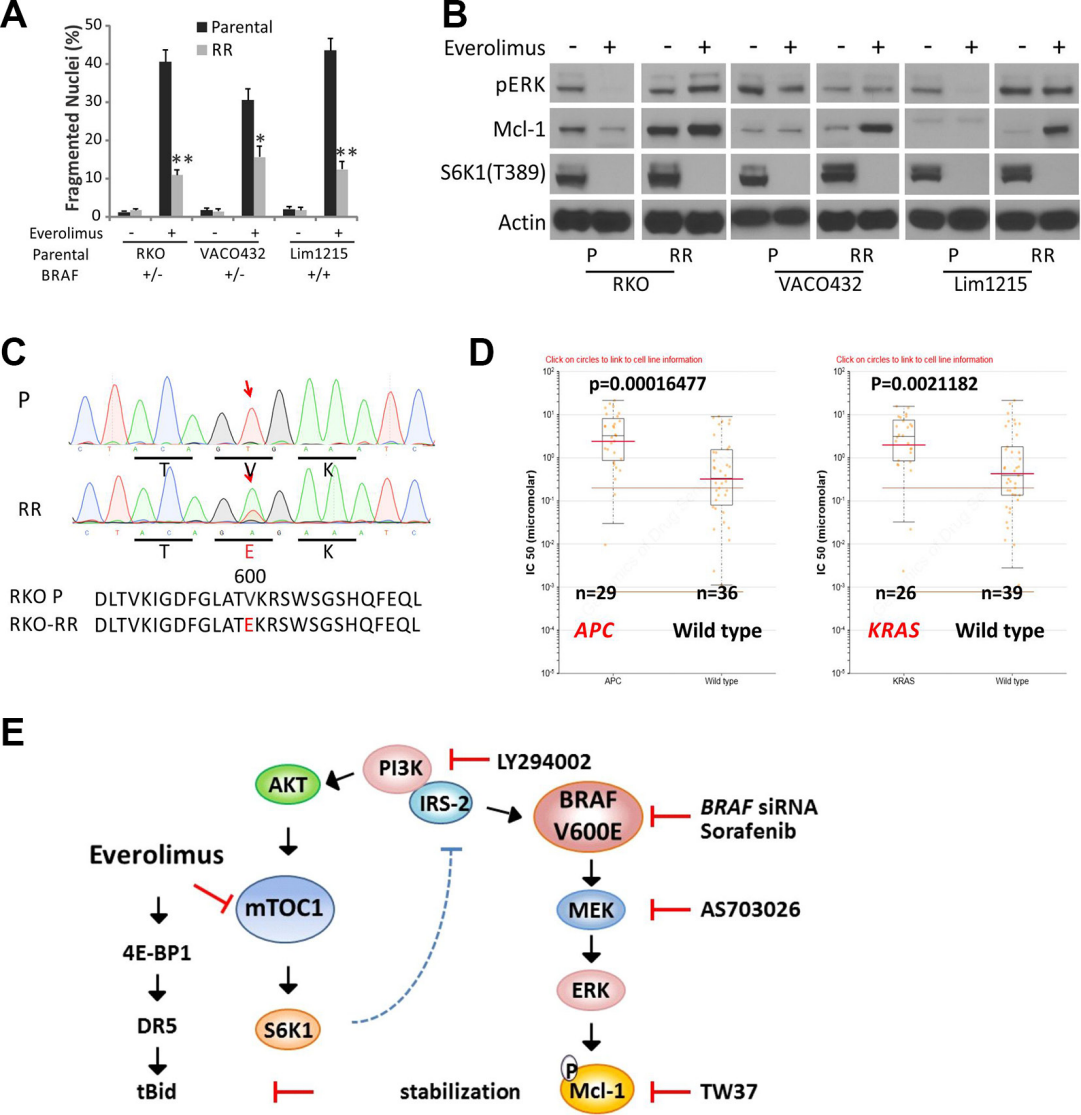
Mcl-1 induction and *BRAF600E* and acquired Everolimus resistance Three parental *BRAF* WT cell lines, RKO (+/−), VACO432 (+/−) Lim1215 (+/+) were subjected to multiple cycles of Everolimus treatment to select for RAD001 resistant (RR) culture. (**A**) the parental and resulting RR cell lines were treated with 20 μM Everolimus for 48 h, and analyzed for apoptosis. **P < 0.01, RR vs. parental culture. (**B**) cells treated as in A for 24 h were analyzed by western blotting. (**C**) detection of *BRAF V600E* in RKO-RR culture that started from *BRAF* WT cells. Red arrows denotes the affected nucleotide and codon. (**D**) *APC* or *KRAS* genotype and Temsirolimus sensitivity in digestive cancer cell lines obtained from Genomics of Drug Sensitivity in Cancer Project. (**E**) a proposed model for Everolimus resistance in *BRAF 600E* cells via ERK-mediated Mcl-1 stabilization that blocks the crosstalk between the death receptor and mitochondrial pathways.

To determine a potential role of *BRAF600E* in acquired resistance to mTOR inhibitors, we established Everolimus-resistant (RR) cultures using three *BRAF* WT cell lines (RKO *BRAF+/−*, VACO +/−, and Lim1215 +/+) through repeated exposure to increasing doses of Everolimus. The resulted cultures were referred as RKO-RR, VACO432-RR and Lim1215-RR. Compared to parental (P) cell lines, RR cultures were highly resistant to Everolimus-induced apoptosis (Figure 6A), and showed strong elevation of Mcl-1 with fully inhibited p-S6K1, while p-ERK elevation was only observed in RKO-RR culture (Figure 6B). Interestingly *BRAFV600E* was only detected in RKO-RR culture (Figure 6C) that was cross-resistant to Torin-1 (Figure S5C).

We further explored the publicly available drug sensitivity data in cell lines from the Genomics of Drug Sensitivity in Cancer Project (Sanger Institute, http://www.cancerrxgene.org), and found that *APC* or *RAS* mutation is an independently predictor of Temsirolimus resistance in digestive cancer cells (Figure 6D), which induces CHOP/DR5-dependent apoptosis in CRC cells similarly as Everolimus as we have shown [19]. Taken together, our data strongly suggest that Mcl-1 upregulation is likely a key resistance mechanism to Everolimus in *BRAF600E* cells (Figure 6E), while other CRC drivers including the gatekeeper *APC* mutation might strongly influence Everolimus sensitivity.

## DISCUSSION

Aberrant activation of RAS/RAF/MEK/ERK and PI3K/AKT/mTOR pathways underlies the hall marks of cancer such as increased cell proliferation, cell motility, angiogenesis, and resistance to apoptosis [30]. Complex compensatory mechanisms between these two pathways and cancer driver mutations strongly influence therapeutic responses of targeted agents [31, 32]. The antitumor activities of rapalogs include inhibition of tumor cell growth, metabolism and angiogenesis [1, 2] as well as induction of apoptosis [19]. In the current study, we identified Mcl-1 upregulation and *BRAF600E* as resistance mechanisms of mTOR inhibitors in colon cancer cells and in xenografts. BRAF600E-dependent activation of MEK/ERK signaling upon the disruption of S6K1-IRS2/PI3K negative feedback stabilizes Mcl-1 to inhibit Bid-mediated crosstalk from the death receptors to mitochondria and apoptosis.

A major challenge in the clinical use of mTOR inhibitors and most targeted therapies is the lack of biomarkers for patient stratification and response monitoring [1, 3]. Resistance mechanisms to rapalogs are complex [2, 6], ranging from compensatory activation of mTORC2/PI3K/AKT [7, 8], ERK/MAPK [27], up-regulation of the PIM family of oncogenic kinases [33], oxidative stress [34], incomplete inhibition of phosphorylation of 4E-BP1 [35], and *mTOR* mutations reducing drug-binding [4]. However, most of these changes are biochemical in nature, and impractical to assess prior to or during treatment. Our work demonstrates *BRAF600E* as a cause of mTOR inhibitor resistance. This finding is significant as DNA-based test coupled with liquid biopsy might provide highly sensitive and specific assays to follow therapeutic responses, where repeated biopsy of solid tumors is impractical or complicated by tumor heterogeneity 36].

Our findings suggest several strategies for overcoming resistance to mTOR inhibitors, and support that Mcl-1 inhibitors or BH3 mimetics [37], PI3K or BRAF inhibitors [38] likely provide a strong synergy to kill Mcl-1 addicted cells or those with *KRAS/BRAF* mutations. Everolimus treatment also induced the expression of several BH3-only proteins such as PUMA, Bim, which likely potentiates apoptosis and Bid-mediated crosstalk to the mitochondria, further supporting the use of BH3 mimetics. However, strong activation of DISC and caspase-8 kills *BRAF600E* cells bypassing Mcl-1 and the mitochondrial pathway, which is achieved by transient exposure to high concentrations of rapalogs or its combination with 5-FU or TRAIL as shown [19].

Using resistant models developed by *in vitro* selection, we showed that Mcl-1 upregulation is likely a key resistance mechanism to Everolimus and ATP-competitive mTOR inhibitor Torin-1, while both *BRAF600E*-dependent and-independent mechanisms might be involved. The *BRAF600E* in RKO-RR culture might rise *de novo* or through the expansion of rare cells in the parental *BRAF* WT culture. It is possible that we missed *BRAF* mutations outside of Exon 15 in the other two cultures, but alternative mechanisms are more likely as elevated p-ERK was not observed. Mcl-1 protein turnover is highly regulated [24]. Alterations in E3 ligase FBW7 [25], deubiquitinase USP9X [26], or upstream regulators such as c-Met, EGFR or KRAS [22, 35] might contribute to Mcl-l upregulation [9]. Though the response of mutant *KRAS* CRC cells is likely to be more context-dependent [39].

Rapalogs induce apoptosis in a variety of tumor models *in vivo*, however most solid tumors including CRC cells are resistant to apoptosis induction or loss of p-4E-BP1 in culture [1–3]. High levels of growth factors or nutrients might lead to “cell culture” resistance, while *APC* or *RAS/RAF* mutation can lead to intrinsic resistance. These mechanisms likely work in concert to prevent activation of destructive ER stress and CHOP/DR5-dependent apoptosis [19]. Complex interactions of oncogenic pathways are clearly influenced by the tissue type, driver mutation and choice of agent [31]. The use of isogenic cell lines and a collection of well characterized cell line models are therefore invaluable in helping model drug actions in preclinical models and guide future clinical testing [20].

In summary, our work identifies EKR-mediated Mcl-1 stabilization via feedback activation of S6K1-IRS-2/PI3K as a novel resistance mechanism to mTOR inhibitors in *BRAF600E* colon cancer cells. Our findings suggest that *BRAF600E* and some *KRAS* mutant colon cancers are unlikely to respond to mono therapy targeting mTOR, but might benefit from combination therapy with BH3 mimetics, or inhibitors of PI3K, Raf or MEK. Further study of these combinations in mouse models or patient-derived xenograft models is warranted.

## MATERIALS AND METHODS

### Cell culture and treatment

The human colorectal cancer cell lines, including Lim2405, RKO, HT29, VACO432, SW1417, Lim1215, Caco-2, and C99, HCA46 and HDC82 were obtained from the American Type Culture Collection (Manassas, VA, USA) and Alberto Bardelli (University of Torino, Italy), respectively. Isogenic RKO and VACO432 *BRAF* WT and V600E mutant cells [21], and isogenic *KRAS* HCT 116, DLD1, Lim1215, and SW48 [21, 23] cell lines were provided by Bert Vogelstein (Howard Hughes Medical Institute, Johns Hopkins University, Baltimore, Maryland, USA) and Alberto Bardelli (University of Torino, Italy) and some through Horizon Discovery (Cambridge, MA, USA). Everolimus-resistant cell lines were generated by treating parental *BRAF* WT cells with increased concentrations of Everolimus from 15 μM to the final 30 μM over a 5-month period with 2 days treatment followed by 5 days of recovery. Exon 15 of *BRAF* was amplified from cDNA and sequenced. Mycoplasma testing was performed routinely by PCR. Cell lines were last tested for the absence of Mycoplasma, genotype, drug response and morphology in August 2015. We examined loss of expression of targeted proteins by western blotting routinely; no additional authentication was done by the authors. Details on cell culture and primers are found in the supplementary materials.

### Western blotting and immunoprecipitation

Western blotting was performed as previously described [40]. For immunoprecipitation, following cell harvest from T-75 flasks, 5 mg of protein reconstituted in 1 ml RIPA buffer and 5 μg of antibody was used for each experiment with Invitrogen Protein A and G Dynabead^®^ immunoprecipitation system according to manufacturer's instructions as previously described [41]. Resulting immunocomplex was analyzed by western blotting. Details on antibodies are found in the supplementary materials.

### Real-time reverse transcriptase (RT) PCR

Total RNA was isolated from cells using the Mini RNA Isolation II Kit (Zymo Research, Orange, CA) according to the manufacturer's protocol. One μg of total RNA was used to generate cDNA using Superscript III reverse transcriptase (Invitrogen, Carlsbad, CA, USA). Real-time PCR was carried out as described [19, 42] Details on primers are found in the supplementary materials (Table S2).

### Analysis of cell viability, apoptosis and cell death

Cell growth was measured by MTS, and apoptosis was analyzed by nuclear staining with Hoechst 33258 (Invitrogen), and Annexin V/propidium iodide (PI) (Invitrogen) followed by flow cytometry as described [43, 44]. For crystal violet assays, the same number of cells were treated for 48 hours in 12-well plates, and attached cells were stained with crystal violet (Sigma, St. Louis, MO) [40]. Details are found in the supplementary materials.

### Transfection and plasmids

The human V5-Mcl-1 expression construct has been previously described [45], and HA-S6K1, HA-S6K1-E389 delta CT [28] expression constructs were obtained from Addgene (Cambridge, MA, USA). Transfection was performed using Lipofectamine 2000 according to the manufacturer's instructions. Mcl-1, Bid, Bim, PUMA, BRAF and IRS-2 small-interfering RNA (siRNA) duplexes were synthesized by Dharmacon (Lafayette, CO, USA). Details for transfection, drug selection and siRNA sequence are found in the supplementary materials (Table S3).

### Xenograft studies

All animal experiments were approved by the University of Pittsburgh Institutional Animal Care and Use Committee. Female 5–6 week-old Nu/Nu mice (Charles River, Wilmington, MA) were housed in a sterile environment with micro isolator cages and allowed access to water and chow *ad libitum*. Mice were injected subcutaneously in both flanks with 4 × 10^6^ WT or *BRAF600E* RKO cells. After implantation, tumors were allowed to grow to 50 mm^3^, approximately 7 days *BRAF 600E* 13 days for *BRAF* WT before treatment was initiated. Mice were randomized into two groups to receive either vehicle or Everolimus (7.5 mg/kg/day) in saline for ten days by oral gavage. Detailed methods on tumor measurements and analysis are found in the Supplementary Materials as described [43, 46]

### Statistical analysis

Statistical analyses were carried out using GraphPad Prism IV software. *P* values were calculated by the student's *t*-test and were considered significant if *p* < 0.05. The means ± one standard deviation (s.d.) are displayed in the figures.

## SUPPLEMENTARY MATERIALS FIGURE AND TABLES



## Abbreviations

ERK: Extracellular signal-regulated kinases
mTOR: mammalian target of rapamycin (mTOR)
S6K1: p70-S6 Kinase 1
IRS-1: Insulin receptor substrate 1
IRS-2: Insulin receptor substrate 2
