# Supplementary material for: BRAFV600E-dependent Mcl-1 stabilization leads to everolimus resistance in colon cancer cells
Source: Oncotarget. 2016 Jun 24;7(30):47699–710. doi: 10.18632/oncotarget.10277 (PMC5216972; doi:10.18632/oncotarget.10277)
Supplement: Supplementary file 1 [file oncotarget-07-47699-s001.pdf]

BRAFV600E-dependent Mcl-1 stabilization leads to everolimus resistance in colon cancer cells

Supplementary Materials

MATERIALS AND METHODS

Cell culture and treatment

The human colorectal cancer cell lines, including Lim2405, RKO, HT29, VACO432, SW1417, Lim1215, Caco-2, and C99, HCA46 and HDC82 were obtained from the American Type Culture Collection (Manassas, VA, USA) and Alberto Bardelli (University of Torino, Italy), respectively. Isogenic RKO and VACO432 WT and V600E *BRAF* mutant cells [1], and isogenic *KRAS* HCT 116, DLD1, Lim1215, and SW48 [2, 3] lines were provided by Bert Vogelstein (Howard Hughes Medical Institute, Johns Hopkins University, Baltimore, Maryland, USA) and Alberto Bardelli (University of Torino, Italy) and some through Horizon Discovery (Cambridge, MA, USA). All cell lines were cultured in McCoy's 5A modified medium (Invitrogen, Carlsbad, CA, USA) supplemented with 10% defined fetal bovine serum (Hyclone, Logan, UT, USA), 100 units/mL penicillin, and 100 µg/mL streptomycin (Invitrogen). Cells were maintained in a 37°C incubator at 5% CO2. For drug treatment, cells were plated in 12-well plates at 20–30% density 24 hr prior to treatment. The DMSO (Sigma, St Louis, MO, USA) stocks of the agents used include Everolimus (LC Laboratories, Woburn, MA, USA) and Torin-1 (Cayman chemical, San Diego, CA), and were diluted into appropriate concentrations with the cell culture medium. Everolimus-resistant cell lines were generated by treating parental *BRAF* WT cells with increased concentrations of Everolimus from 15 µM to the final 30 µM over a 5-month period with 2 days treatment followed by 5 days of recovery. Cell lines were last tested for the absence of *Mycoplasma*, genotype, drug response and morphology in August 2015. We examined loss of expression of targeted proteins by western blotting routinely; no additional authentication was done by the authors.

Cell viability

Cells were plated at 20–30% confluence 24 hours prior to treatment. Unless noted otherwise, cells were treated for 48 hours. Apoptosis was analyzed by nuclear fragmentation assay with 300 or more cells scored for each determination, and by flow cytometry (50,000 events) [4, 5]. Cell proliferation was measured using Cell-Titer 96 Aqueous One Solution Cell Proliferation Assay (MTS assay), according to manufacturers with recommendations. Triplicates were used in each condition, and experiments were repeated for at least 3 times.

Western blotting

Western blotting was performed as previously described [6]. The following antibodies were used: Mcl-1 (Cat#559027, BD, San Jose, CA, USA), Bax (Cat#610983, BD), Bak (Cat#06536, Millipore, Bellerica, MA, USA), Noxa (Cat# OP180, Millipore), Bid (Cat#2002, Cell Signaling, Danvers, MA, USA), AKT (S473) (Cat#4058, Cell Signaling), Mcl-1 (Ser159/Thr163) (Cat# 4579, Cell Signaling), ERK1/2 (T203/204) (Cat#4376, Cell Signaling), 70S6K1(T389) (Cat#9234, Cell Signaling), RPS6 (Ser235/236) (Cat#4858, Cell Signaling), active caspase-3 (Cat#9661, Cell Signaling), 4E-BP1 (S65/70) (Cat#9451, Cell Signaling), eiF2a (S51) (Cat# 3398, Cell Signaling), IRS1 (Cat#3407, Cell Signaling), IRS-2 (Cat#4502, Cell Signaling), DR5(Cat#3696, Cell signaling), CHOP (Cat#2895, Cell Signaling), B-Raf (Cat #14814, Cell signaling), PUMA [7] and β-actin (Cat#A5441, Sigma).

Transfection

Small-interfering RNA (siRNA) duplexes, and control scrambled siRNA were synthesized from Dharmacon (Lafayette, CO, USA). Cells were transfected with 400 pmols of siRNA duplexes/well in 12-well plates for 4 hours, followed by incubation in medium containing 5% FBS for 20 hours, and then followed by drug treatment in complete media containing 10% FBS. Cells were transfected in 12-well plates with 0.8 ug/well of expressing plasmid for 4 hours followed by 20 hours of recovery prior to drug treatment. Results are pooled from or average of three-independent wells in a representative experiment, and repeated at least twice with similar results.

Xenograft studies

Tumor diameters were measured every other day, and tumor volumes were calculated ($width^2 \times length \times 0.5$). The averaged tumor volumes of each group were plotted with one standard deviation, and *P*-value was calculated on the final measurements. Mice were

euthanized when tumors reached ~ 800m^3 in size. Tumors were dissected and fixed in 10% formalin before paraffin embedding. For apoptosis and western blot analysis, two randomly selected tumor bearing mice were sacrificed to harvest tumors on day 11 for analysis as described [8]. Quantitation was based on sections from two randomly chosen tumors in each group, and scoring of 400 or more cells/tumor in high power fields (400x). Active caspase-3, Terminal deoxynucleotidyl transferase dUTP nick -end labeling (TUNEL), p-ERK, eiF2a (S51), 4E-BP1 (S65/70) immunostaining were performed on 5 μM paraffin-embedded tumor sections with HRP (Pierce)- or Alexa Fluor488-conjugated secondary antibodies (Invitrogen) for signal detection.

REFERENCES

1. Yun J, Rago C, Cheong I, Pagliarini R, Angenendt P, Rajagopalan H, Schmidt K, Willson JK, Markowitz S, Zhou S, Diaz LA Jr, Velculescu VE, Lengauer C, et al. Glucose deprivation contributes to the development of KRAS pathway mutations in tumor cells. Science. 2009; 325:1555–1559.
2. Di Nicolantonio F, Arena S, Tabernero J, Grosso S, Molinari F, Macarulla T, Russo M, Cancelliere C, Zecchin D, Mazzucchelli L, Sasazuki T, Shirasawa S, Geuna M, et al. Deregulation of the PI3K and KRAS signaling pathways in human cancer cells determines their response to everolimus. J Clin Invest. 2010; 120:2858–2866.
3. Misale S, Yaeger R, Hobor S, Scala E, Janakiraman M, Liska D, Valtorta E, Schiavo R, Buscarino M, Siravegna G, Bencardino K, Cercek A, Chen CT, et al. Emergence of KRAS mutations and acquired resistance to anti-EGFR therapy in colorectal cancer. Nature. 2012; 486:532–536.
4. Yu J, Yue W, Wu B, Zhang L. PUMA sensitizes lung cancer cells to chemotherapeutic agents and irradiation. Clin Cancer Res. 2006; 12:2928–2936.
5. Sun Q, Sakaida T, Yue W, Gollin SM, Yu J. Chemosensitization of head and neck cancer cells by PUMA. Mol Cancer Ther. 2007; 6:3180–3188.
6. Yu J, Zhang L, Hwang PM, Kinzler KW, Vogelstein B. PUMA induces the rapid apoptosis of colorectal cancer cells. Mol Cell. 2001; 7:673–682.
7. Yu J, Wang Z, Kinzler KW, Vogelstein B, Zhang L. PUMA mediates the apoptotic response to p53 in colorectal cancer cells. Proc Natl Acad Sci USA. 2003; 100:1931–1936.
8. Yu J, Yue W, Wu B, Zhang L. PUMA sensitizes lung cancer cells to chemotherapeutic agents and irradiation. Clin Cancer Res. 2006; 12:2928–2936.
9. Mouradov D, Sloggett C, Jorissen RN, Love CG, Li S, Burgess AW, Arango D, Strausberg RL, Buchanan D, Wormald S, O'Connor L, Wilding JL, Bicknell D, et al. Colorectal cancer cell lines are representative models of the main molecular subtypes of primary cancer. Cancer Res. 2014; 74:3238–3247.
10. Ming L, Sakaida T, Yue W, Jha A, Zhang L, Yu J. Sp1 and p73 Activate PUMA Following Serum Starvation. Carcinogenesis. 2008; 29:1878–1884.

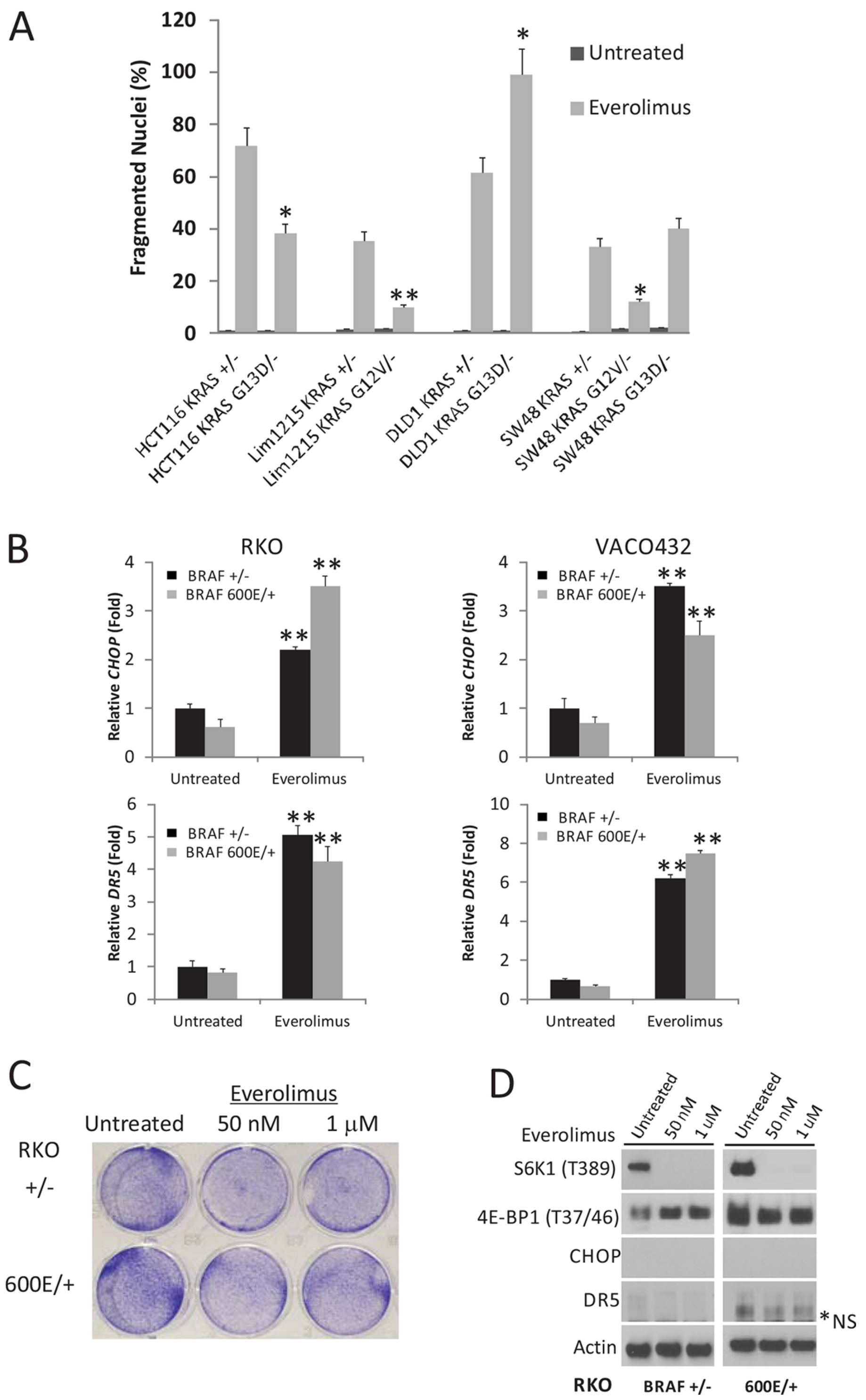

Supplementary Figure S1:Comparable levels of ER stress and DR5 induction by Everolimus in *BRAF* WT and V600E cells. (**A**) Four sets of isogenic cell lines with WT or mutant KRAS were treated with Everolimus (20 μM for SW48 and 25 μM for all other) for 48 h, and analyzed for apoptosis by counting condensed and fragmented nuclei. **$P < 0.01$, *$P < 0.05$, Mutant *vs*. WT *KRAS*. (**B**) isogenic cells were treated with Everolimus for 24 h and analyzed for the expression CHOP and DR5 by real-time RT-PCR. **$P < 0.01$, Everolimus vs. untreated. (**C**) isogenic RKO cells were treated with low concentrations of Everolimus (50 nM and 1 μM) for 48 h, and attached cells were stained by crystal violet. (**D**) cells were treated as in B for 24 h, and analyzed by western blotting. *NS, the lower and non-specific bands.

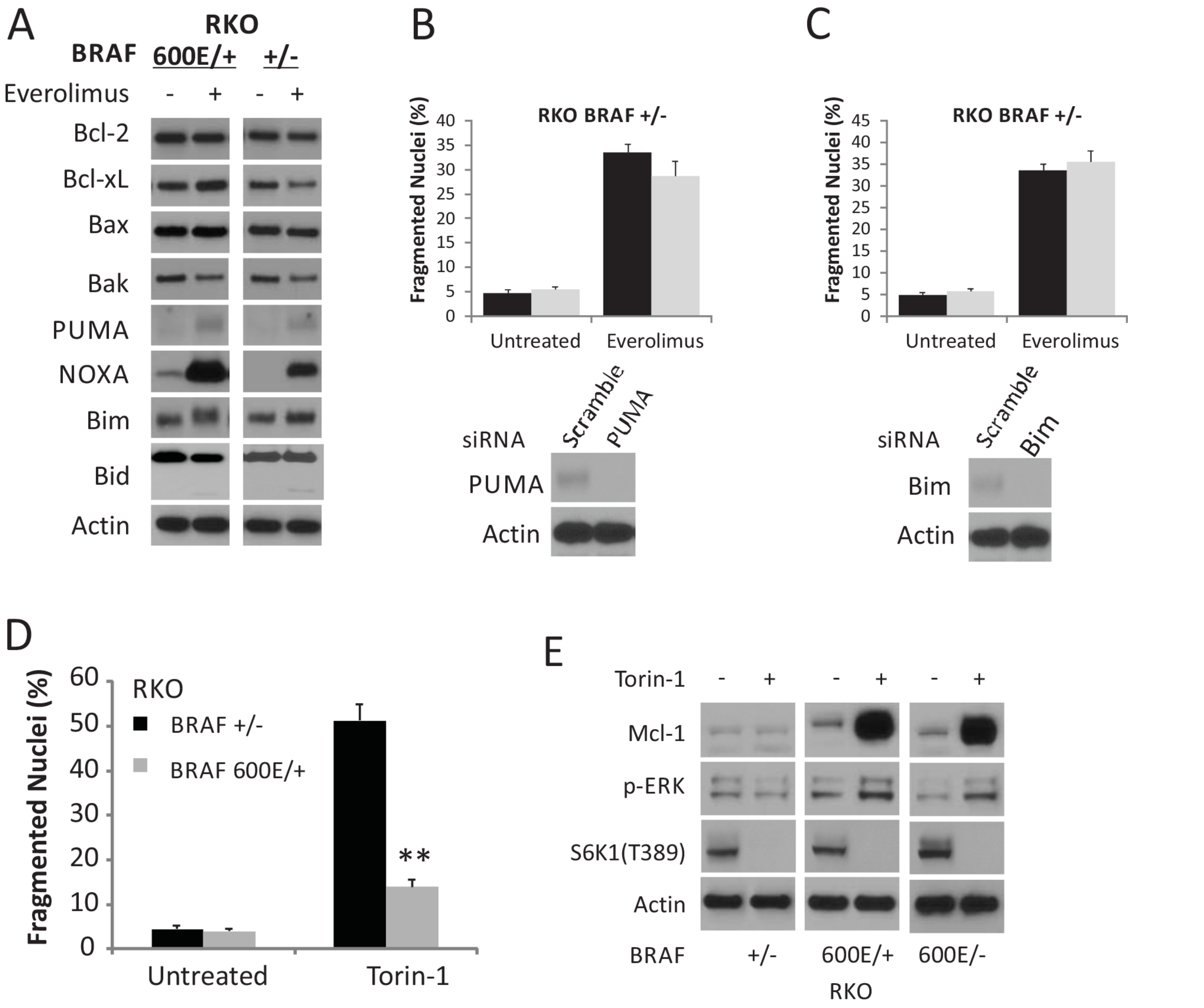

Supplementary Figure S2: Mcl-1 induction blocks Bid-mediated crosstalk with the mitochondrial apoptotic pathway in *BRAF 600E* cells. (**A**) isogenic RKO cells were treated with 20 μM Everolimus for 24 h, and analyzed by western blotting. (**B** and **C**) *BRAF* WT (+/−) RKO cells (bottom) were transfected by either a scrambled, PUMA (B) or Bid (C) siRNA for 24 h, then treated with Everolimus for 48 h, and analyzed for apoptosis. (**D**) isogenic RKO cells were treated with 15 μM Torin-1 for 48 h and analyzed for apoptosis. ***P* < 0.01, 600E vs. WT *BRAF*. (**E**) cells treated as in D for 24 h were analyzed by western blotting. β-Actin was used as a loading control.

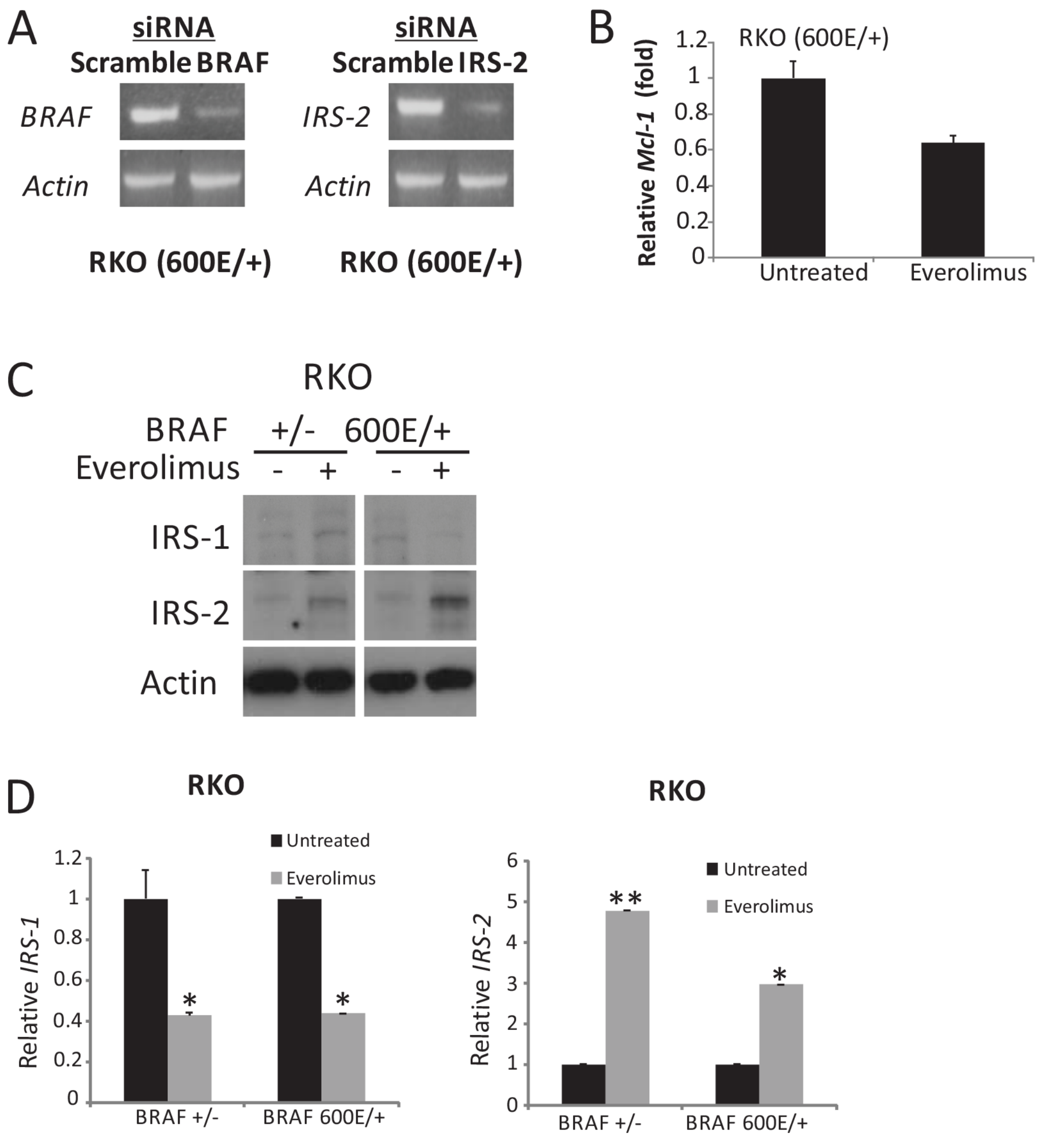

Supplementary Figure S3: mRNA levels of selected targets after Everolimus treatment. (**A**) *BRAF* and *IRS-2* knockdown by siRNA was confirmed by RT-PCR. (**B**) *Mcl-1* mRNA was analyzed by realtime RT-PCR in BRAF 600E RKO cells 24 h after Everolimus treatment. (**C**) isogenic RKO cells treated with Everolimus for 24 h, and analyzed for IRS-1 and IRS-2 by western blotting. (**D**) *IRS-1* and *IRS-2* mRNA was analyzed by realtime RT-PCR in *BRAF* WT and 600E RKO cells 24 h after Everolimus treatment. $^{**}P < 0.01$, $^{*}P < 0.05$, Everolimus *vs.* untreated.

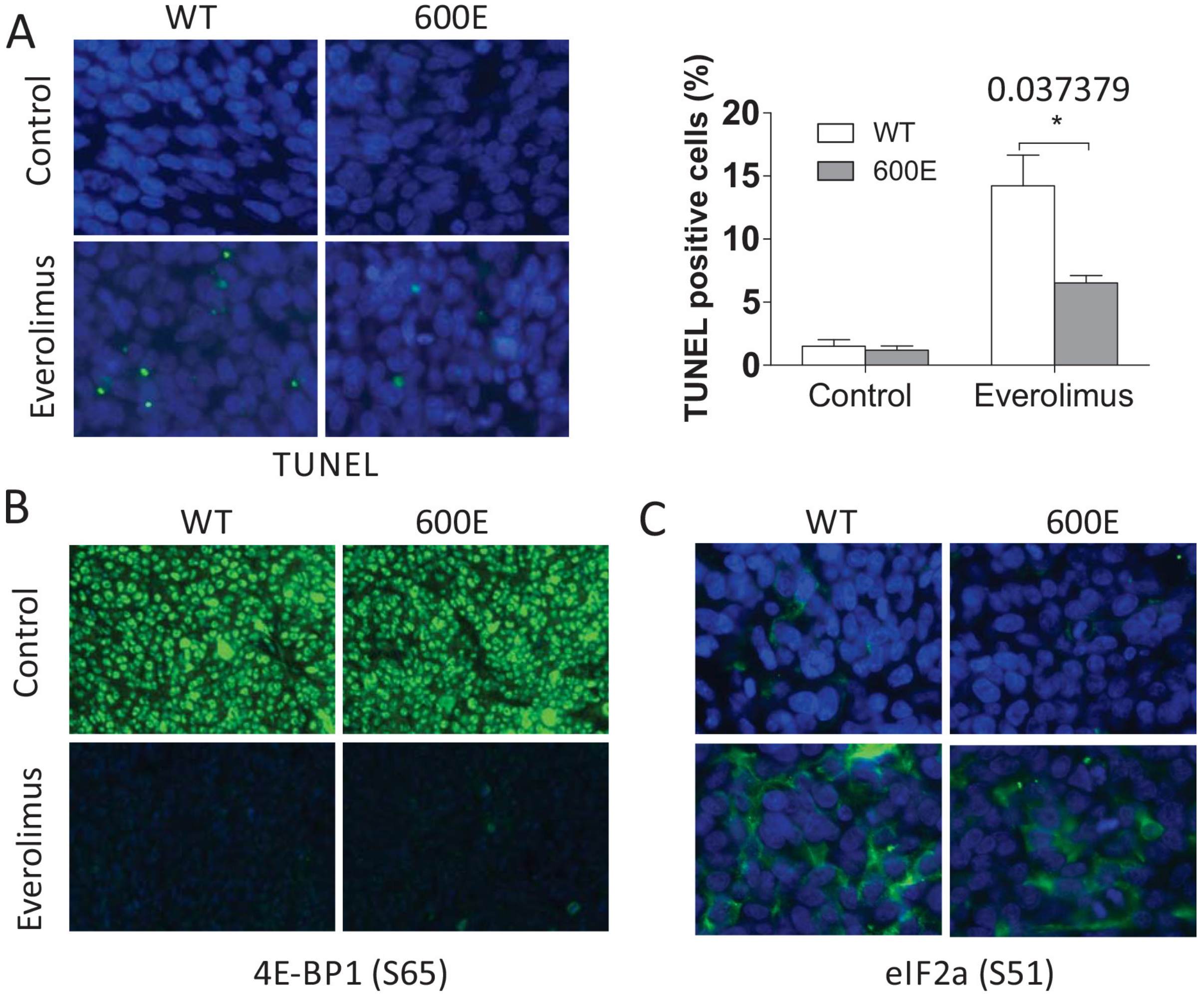

Supplementary Figure S4: Induction of apoptosis and ER stress in xenograft tumors after Everolimus treatment. (**A**) representative images of TUNEL staining in tumors (left) with quantitation (right). **P* < 0.05, 600E *vs*. WT *BRAF*. *P* value was calculated using Student's *t*-test, two tailed. (**B**) representative images of 4E-BP1 (S65), and CeIF2a (S51) staining in tumors.

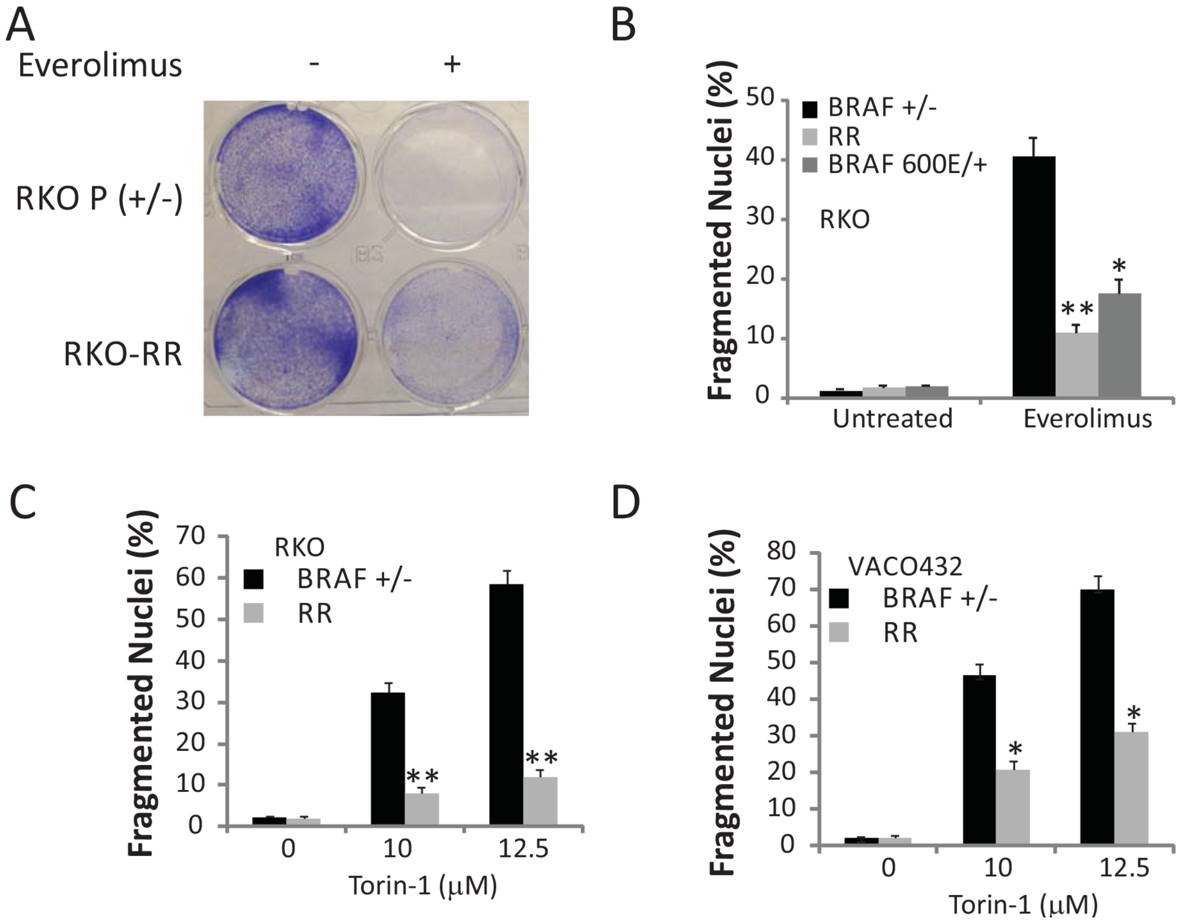

Supplementary Figure S5: Characterization of *in vitro* selected Everolimus-resistant cultures. (**A**) *BRAF* WT (+/−) RKO and –RR were treated with 20 μM Everolimus, attached cells after 48 h were stained by crystal violet. (**B**) BRAF WT (+/−), selected RR, or BRAF 600E RKO cells were treated as in A for 48 h and analyzed for apoptosis. (**C**) BRAF WT (+/−) RKO and –RR were treated with Torin-1 for 48 h and analyzed for apoptosis. (**D**) BRAF WT (+/−) VACO432 and –RR were treated with Torin-1 for 48 h and analyzed for apoptosis. $^{**}P < 0.01$, $^{*}P < 0.05$, BRAF 600E, RR culture *vs.* WT *BRAF*.

Supplementary Table S1: Colon rectal cancer cell lines used in this study with known driver mutations

Cell Line	BRAF	KRAS	PIK3CA	APC	β-catenin	TP53	SMAD4
Lim2405	mut	wt	wt	mut	wt	wt	wt
RKO	mut	wt	mut	wt	mut	wt	wt
HT29	mut	wt	mut	mut	wt	mut	mut
VACO432	mut	wt	mut	mut	wt	mut	wt
SW1417	mut	wt	wt	mut	wt	mut	wt
C99	wt	wt	wt	wt	wt	wt	wt
HCA46	wt	wt	mut	mut	wt	mut	wt
Lim1215	wt	wt	wt	wt	mut	wt	wt
Caco2	wt	wt	wt	mut	mut	mut	mut
HDC82	wt	wt	wt	mut	wt	mut	wt
HCT 116	wt	mut	mut	wt	mut	wt	wt
DLD1	wt	mut	mut	mut	wt	wt	wt
SW48	wt	wt	mut	mut	mut	wt	wt

Information has been compiled from [1, 9].

Supplementary Table S2: Primers used in this study for RT-PCR and sequencing analysis

Gene	Primer	Sequence	purpose
**Mcl-1**	**899F**	5′-ATGCTTCGGAAACTGGACAT-3′	RT-PCR
	**1167R**	5′-TGGAAGAACTCCACAAACCCA-3′	RT-PCR
**BRAF**	**1158F**	5′-AGGATTTCGTGGTGATGGAG-3′	RT-PCR & sequence
	**2016R**	5′-TCCTCGTCCCACCATAAAAA-3′	RT-PCR & sequence
**IRS-1**	**Forward**	5′-GTTTCCAGAAGCAGCCAGAG-3′	RT-PCR
	**Reverse**	5′-ACTCTCTCCACCCAAGGTGA-3′	RT-PCR
**IRS2**	**Forward**	5′-CCACCATCGTGAAAGAGTGA-3′	RT-PCR
	**Reverse**	5′-AACAAGGGAAAGAGGCAGGT-3′	RT-PCR
**DR5**	**Forward**	5′-AAGACCCTTGTGCTCGTTGT-3′	RT-PCR
	**Reverse**	5′-AGGTGGACACAATCCCTCTG-3′	RT-PCR
**CHOP**	**Forward**	5′-TGGAAATGAAGAGGAAGAATCAAAA-3′	RT-PCR
	**Reverse**	5′-CTTGGTGCAGATTCACCATTC-3′	RT-PCR

Supplementary Table S3: siRNAs used in this study

Gene	siRNA	Sequence
**Mcl-1**	**Mcl-1-956**	5′-CGCCGAATTCATTAATTTA(dT)-3′
**BRAF**	**BRAF**	5′-AAGCACGCTTAGATTGGAATA(dT)-3
**IRS-2**	**IRS-2**	5′-CCAAGCACAAGTACCTGAT(dTdT)-3′
**Bid**	**Bid-238**	5′-CCCGCCGAATTCATTAATTTA(dT)-3′
**Bim**	**Bim-27**	5′-GACCGAGAAGGTAGACAAT(dTdT)-3′
**PUMA**	**PUMA-721**	5′-ACCTCAACGCAATACGA(dTdT)-3′
	**PUMA-1559**	5′-ACGTGTGACCACTGGCATT(dTdT)-3′

The corresponding sequence in the cDNA is shown. Overhangs are indicated with (). Two siRNAs were used for PUMA knockdown [10].
